# Tao-Hong-Si-Wu-Tang protects against methionine- and choline-deficient diet-induced hepatic steatosis in rats and is associated with inhibition of oxidative stress, inflammation, apoptosis, and pyroptosis

**DOI:** 10.3389/fimmu.2026.1767119

**Published:** 2026-07-06

**Authors:** Luyao Xu, Qiuling Li, Chengqin Lu, Mincheng Zheng, Wenhai Guo, Yun Chen, Jiaying Dai, Yao Deng, Ning Zhang, Kanlin Yang, Xinling Chen, Kangrong Wang, Guohang Yan, Hantao Li, Rui Duan, Jiean Xu, Wei Liu, Hongzhi Yang, Jinwen Xu, Min Dai, Yaxing Zhang

**Affiliations:** 1 Department of Gynecology, The Second Clinical School of Guangzhou University of Chinese Medicine, Guangzhou University of Chinese Medicine, Guangzhou, Guangdong, China; 2 Department of Physiology, School of Basic Medical Sciences, Guangzhou University of Chinese Medicine, Guangzhou, Guangdong, China; 3 Research Centre of Basic Integrative Medicine, School of Basic Medical Sciences, Guangzhou University of Chinese Medicine, Guangzhou, Guangdong, China; 4 Department of Traditional Chinese Medicine, The Third Affiliated Hospital, Sun Yat-Sen University, Guangzhou, Guangdong, China; 5 Department of Biotechnology Class of 2019, School of Basic Medical Sciences, Guangzhou University of Chinese Medicine, Guangzhou, Guangdong, China; 6 Institute of Integrated Traditional Chinese and Western Medicine, The Third Affiliated Hospital, Sun Yat-sen University, Guangzhou, Guangdong, China; 7 Department of Allergy and Immunology, The Third Affiliated Hospital, Sun Yat-sen University, Guangzhou, Guangdong, China

**Keywords:** GSDMD, GSDME, NAFLD, oxidative stress, pyroptosis, Tao-Hong-Si-Wu-Tang

## Abstract

**Background:**

Non-alcoholic fatty liver disease (NAFLD), which is the most prevalent chronic liver disease, affects more than 30% of the adult population worldwide. Lifestyle interventions and management of metabolic comorbidities are the current main treatments. Thus, it is urgent to find candidate drugs for NAFLD. Traditional Chinese medicine (TCM) formulas have been extensively applied to treat NAFLD in China. Tao-Hong-Si-Wu-Tang (THSWT), a classic TCM formula with anti-oxidation, anti-inflammation, and anti-apoptosis, has emerged as an important formula for hepatoprotection. Based on its biological actions, we speculate that THSWT may have the therapeutic effect on NAFLD.

**Methods:**

For dose exploration, the rats were randomly divided into six groups (n = 3–4 in each group): a control group, a model group, and four treatment groups receiving different doses of THSWT (0.5 g/kg, 2.5 g/kg, 5.0 g/kg, 7.5 g/kg; gavage). NAFLD rat model was established by feeding a methionine- and choline-deficient (MCD) diet for two weeks. On day 8 of MCD diet feeding, THSWT treatment was initiated and continued for seven days, with tissue collection performed on day 15. The effect of THSWT on MCD diet-induced hepatic steatosis was evaluated by haematoxylin and eosin (H&E) staining and hepatic steatosis grade scores. The best dosage of THSWT was used to further confirm the protection of THSWT on MCD diet-induced hepatic steatosis in rats. Therefore, a new batch of animals was randomly divided into three groups: a control group, a model group, and a THSWT treatment group (n = 7 in each group). The MCD modelling and THSWT treatment protocols were the same as above. H&E, Oil Red O, and Masson’s trichrome staining, qPCR for lipid metabolism-related genes, immunoblot for Collagen I and II, and the commercially available kits for hepatic triglycerides (TG) and hydroxyproline (HYP) and serum alanine aminotransferase (ALT) and aspartate aminotransferase (AST) levels were used to evaluate the effects of THSWT on hepatic steatosis. Furthermore, immunofluorescence staining and immunoblot were performed to examine the key proteins expression and activation involved in the protection of THSWT on NAFLD.

**Results:**

THSWT (2.5 g/kg/day) treatment can alleviate MCD diet-induced hepatic steatosis (including slight fibrosis) in rats. Mechanistically, THSWT treatment is associated with diminishing hepatic reactive oxygen species (ROS), 3-nitrotyrosine (3-NT) and malondialdehyde (MDA) levels, while increasing hepatic GSH levels; inhibiting TLR4/9-IKK-NF-κB-TNF-α/IL-1β/IL-18 innate immune signalling; suppressing JNK and Caspase-3 apoptosis signalling, and Caspase-1/11/8-Gasdermin D (GSDMD) and Caspase-3-GSDME pyroptosis signalling in the liver of rats feeding a MCD diet.

**Conclusions:**

THSWT alleviated MCD diet-induced hepatic steatosis in rats possibly *via* inhibiting hepatic oxidative stress, pro-inflammatory cytokines expression, apoptosis, and pyroptosis. THSWT is a classic prescription worth exploring for treating hepatic steatosis in future.

## 1 Introduction

Non-alcoholic fatty liver disease (NAFLD; now known as metabolic dysfunction-associated steatotic liver disease, MASLD) is defined as fat accumulation in at least 5% of hepatocytes in the absence of secondary causes of lipid accumulation, such as significant alcohol consumption (daily intake <30 g in men and <20 g in women), long-term use of a steatogenic medication, or monogenic hereditary disorders (1, 2). NAFLD encompasses a spectrum of diseases, extending from simple steatosis (referred to as non-alcoholic fatty liver, NAFL) to steatosis accompanied by inflammation and ballooning hepatocyte cell death termed non-alcoholic steatohepatitis (NASH), which potentially progresses to hepatic fibrosis, cirrhosis, and even hepatocellular carcinoma (HCC) (1, 3, 4). The global prevalence of NAFLD is approximately 30% to 40% in the general adult population (with varying prevalence across continents), and the national prevalence of NAFLD in China was approximately 30.4% (5, 6). Furthermore, NAFLD pandemic fuels an upsurge in cardiovascular diseases (CVDs), which is one of the leading causes of death in patients with NAFLD (3, 7, 8). Therefore, it is urgent to find the candidate drugs for NAFLD.

The multiple and complex signalling, including redox signalling (9), germline-encoded pattern recognition receptors [PRRs, e.g., TLR4 (10), TLR9 (11), and NLRP3 (12, 13)]-mediated innate immune signalling, pro-inflammatory cytokines signalling [e.g., tumour necrosis factor-alpha (TNF-α), interleukin-6 (IL-6), IL-1β, and IL-18 (14–16)], and cell death [e.g., apoptosis (17) and pyroptosis (18, 19)], is characterized by crosstalk and is involved in the pathogenesis of NAFLD/NASH (20–22). Thus, it is significant for treating NAFLD by multiple targets.

The ancient classic traditional Chinese medicine (TCM) formulas have received increasing attention from the medical community and clinical doctors due to their multi-component and multi-target properties. Based on the basic TCM theory, deficiency of blood (血虚) is one of the key mechanisms of NAFLD, and thus, the method of blood-building (Chinese Medical Concept: Bu-Xue/补血) has been used to improve hepatic steatosis (2, 23). Tao-Hong-Si-Wu-Tang (THSWT, 桃红四物汤, which is composed of Shudi (熟地), Chuanxiong (川芎), Baishao (白芍), Danggui (当归), Taoren (桃仁), and Honghua (红花)) is a classic TCM formula with the actions of tonifying blood (补血) and circulating blood (活血). THSWT was first recorded in *Yi-Zong-Jin-Jian* (医宗金鉴, an official TCM classics in China Qing [Qianlong] Dynasty). THSWT is traditionally used for women presenting with early menstruation, menorrhagia, dark purple in colour and viscous in consistency, with the presence of clots, accompanied by abdominal pain and bloating. Now, THSWT, with anti-oxidation, anti-inflammation, and anti-apoptosis actions (24, 25), has emerged as an important formula for hepatoprotection (26–30). Based on its classical TCM actions and modern biological actions, we speculate that THSWT may have the therapeutic effect on diet-induced NAFLD. Therefore, we used methionine- and choline-deficiency (MCD) diet-fed rats to investigate the effect of THSWT on hepatic steatosis and its underlying innate immune mechanisms.

## 2 Materials and methods

### 2.1 Preparation of Tao-Hong-Si-Wu-Tang

The composition of THSWT was according to the Textbook of Fang-Ji-Xue (Shanghai Scientific & Technical Publishers, first edition, ISBN: 978-7-5323-0500-1/R·139). THSWT includes Shudi (*Rehmannia glutinosa* (Gaertn.) Libosch., 15g), Chuanxiong (*Ligusticum chuanxiong* Hort., 8g), Baishao (*Paeonia lactiflora* Pall., 10g), Danggui (*Angelica sinensis* (Oliv.) Diels., 12g), Taoren (*Prunus persica* (Linn) Batsch., 6g), and Honghua (*Carthamus tinctorius* L., 4g). The plant materials prepared in ready-to-use forms were purchased from DaShenLin Pharmaceutical Group Co., Ltd. (Guangzhou, China).

The dried prescriptions of THSWT (55 g) were soaked in 300 mL distilled water for 60 minutes. The formula was initially boiled over high heat and then simmered for 30 minutes over low heat. The first decoction of THSWT was obtained and filtered using an 80-mesh sieve. The same method with an equal volume of distilled water was used to prepare the second decoction. Both the first and second decoctions were combined and concentrated by boiling to a final volume of 110 mL, and then, the concentration of THSWT solution was 0.50 g/mL (dried herb weight/solution). The solution of THSWT was prepared every 3 days and stored at 4 °C before use.

### 2.2 Qualitative analysis of Tao-Hong-Si-Wu-Tang

THSWT water decoction (0.50 g/mL) was diluted tenfold with ultrapure water and centrifuged at 12, 000 rpm for 15 minutes and collected the resulting supernatant. Then, the reference standards were weighted as follows: 10.20 mg Rehmannioside D (Chengdu Must Biological Technology Co., Ltd, MUST-22051017, Chengdu, China), 10.50 mg Hydroxysafflor yellow A (Chengdu Alfa Biotechnology Co., Ltd., AB0241-0020, Chengdu, China), 10.00 mg Amygdalin (Chengdu Alfa Biotechnology Co., Ltd., AB1129-0020, Chengdu, China), 10.30 mg Ferulic acid (Chengdu Alfa Biotechnology Co., Ltd., AB1754-0020, Chengdu, China). 10.50 mg Paeoniflorin (Chengdu Alfa Biotechnology Co., Ltd., AB1259-0020, Chengdu, China). They were each dissolved in 50% (v/v) methanol to prepare individual standard solutions as 51.00 μg/mL, 52.50 μg/mL, 50.00 μg/mL, 51.50 μg/mL, 52.50 μg/mL, respectively. Ultra-Performance Liquid Chromatography – Quadrupole Time-of-Flight Mass Spectrometry (UPLC-Q-TOF-MS; Waters H Class Plus and X500R Q-TOF) and chromatographic column (Waters, ACQUITY UPLC BEH C18, 2.1×100 mm, 1.7 µm) were used to quantify indicative components in THSWT water decoction using a single-point external standard method (the injection volume, 1.00 μL; the dilution factor: 10.00) following standard operating procedures. Meanwhile, the above five reference standards were individually determined for method validation and calculation of the analyte contents in samples.

### 2.3 The hepatic steatosis rat model and Tao-Hong-Si-Wu-Tang treatment

Male Wistar rats were bought from Experimental Animal Management Centre of Southern Medical University (Guangzhou, China). The animals were housed in a temperature-controlled animal facility with a 12-hour light-dark cycle and allowed to obtain rodent chow and water ad libitum. All animal procedures were approved by Institutional Animal Care and Use Committee of Guangzhou University of Chinese Medicine. Animals were randomized using a random number table, with the allocation sequence concealed in sequentially numbered, sealed, opaque envelopes prepared by an investigator not involved in the study.

**Figure 1:**
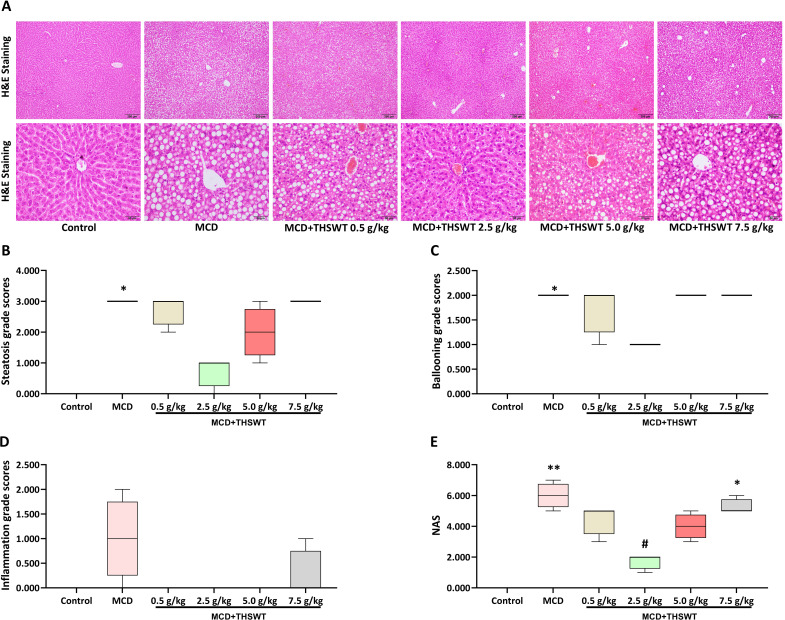
Effects of different dosages of THSWT on hepatic steatosis induced by MCD in rats. H&E staining **(A)** and hepatic steatosis grade scores **(B)**, ballooning grade scores **(C)**, inflammation grade scores **(D)**, and NAFLD activity scores (NAS) **(E)**. n = 3 ~ 4 rats in each group. The data were expressed as median (P25, P75). *p < 0.05 vs control group; **p < 0.01 vs control group; ^#^*p* < 0.05 vs MCD group.

Total 23 Wistar rats (male, 350 to 380 g) were randomly divided into six groups: Control group (n = 3), Model (MCD) group (n = 4), 0.50 g/kg THSWT treatment group (n = 4), 2.50 g/kg THSWT treatment group (n = 4), 5.00 g/kg THSWT treatment group (n = 4), 7.50 g/kg THSWT treatment group (n = 4). Wistar rats were fed with a MCD diet (MD12052, Medicience Ltd., Yangzhou, China) for two weeks to induce hepatic steatosis (31). The rats in Control group were fed with an identical diet sufficient in methionine and choline (MCD control; MD12051, Medicience Ltd., Yangzhou, China) for two weeks. On day 8, different doses of THSWT solution were administered to four treatment groups *via* gavage at 16:00-17:00 pm for seven consecutive days. On day 15, animals were deeply anesthetized for blood collection and euthanized *via* cervical dislocation for collecting liver samples. The liver samples from all animals were subjected to haematoxylin and eosin (H&E) staining followed by assessing hepatic steatosis grade scores (see details in 2.4). H&E staining showed that MCD diet feeding induced the accumulation of lipid droplets in the liver, increased steatosis grade scores, ballooning grade scores, and NAFLD activity scores (NAS), while the 2.5 g/kg dosage of THSWT decreased NAS (**Figure 1**). Therefore, 2.5 g/kg dosage of THSWT was used for further investigation.

In order to further confirm the therapeutic efficacy and its underlying mechanism, total 21 animals were randomly divided into three groups: Control group (n = 7), MCD group (n = 7), and THSWT treatment group (n = 7). The protocol for establishing hepatic steatosis model and treatment was the same as described above. On day 15, animals were deeply anesthetized for blood collection and euthanized *via* cervical dislocation for collecting liver samples. The liver samples from all animals were subjected to H&E staining followed by assessing hepatic steatosis grade scores (see details in 2.4), and measurement of triglycerides (TG), malondialdehyde (MDA), glutathione (GSH), and myeloperoxidase (MPO) levels (see details in 2.5). Blood samples from all animals were used for serum biochemical analysis (see details in 2.6). The samples for other assays were randomly collected, where “n” represents the number of biological replicates.

### 2.4 Histopathology analysis

The fresh liver samples were fixed in a 4% paraformaldehyde solution (Cat# G1101, Servicebio, Wuhan, China) for subsequent haematoxylin (Cat# G1004, Servicebio, Wuhan, China) and eosin (Cat# G1001, Servicebio, Wuhan, China) staining and oil red O (Cat# G1015, Servicebio, Wuhan, China) staining, and Masson’s trichrome (Cat# G1340, Solarbio, Beijing, China) staining as we have previously described (2, 32).

NAFLD activity scores (NAS), defined as the unweighted sum of the scores for steatosis (0-3), lobular inflammation (0-3), and ballooning (0-2), were performed by Chengqin Lu and Guohang Yan blinded to the treatment groups (33, 34).

### 2.5 Hepatic TG, MDA, GSH, MPO and HYP levels analysis

Hepatic TG, MDA, GSH, MPO, and hydroxyproline (HYP) levels were examined by the commercial kits from Nanjing Jiancheng Bioengineering Institute, Nanjing, China (TG, A110-1-1; MDA, A003-1-2; GSH, A006-2-1; MPO, A044-1-1; HYP, A030-2-1) according to the manufacturer’s instructions. Hepatic TG levels were expressed as mmol/g of fresh weight. Hepatic MDA, GSH, MPO, and HYP levels were expressed as nmol/mg and μmol/g of protein, U/g of fresh weight, and μg/mg tissue, respectively. To eliminate the impact of repeated freeze-thaw cycles on GSH levels, all measured GSH values were normalized to the average level of the Control group.

### 2.6 Serum biochemical analysis

The blood samples were collected from the abdominal aorta for separating serum (3, 000 rpm for 15 minutes). Subsequently, the obtained serum was stored at -80˚C until further analysis. The levels of alanine aminotransferase (ALT), aspartate aminotransferase (AST), total cholesterol (TC), and TG in the serum were determined following our previously described protocols (35).

### 2.7 Immunofluorescence staining

The fresh liver tissues were immediately immersed in liquid nitrogen and snap frozen, followed by cryosectioning to assess liver ROS levels by immunofluorescence staining using MitoSOX kit (Cat# M36008, Thermo Fisher Scientific, Waltham, Massachusetts, United States). The working solution of MitoSOX consisted 13 mL Hank’s buffer solution (Cat# H1025, Solarbio, Beijing, China), 13 μL dimethyl sulfoxide (DMSO, Guangzhou Chemical Reagent Factory, 20180501-1), and 50 μg MitoSOX stock solution. The sections were incubated in the working solution at a temperature of 37˚C in a biochemistry cultivation cabinet for 10 minutes and then washed with Hank’s buffer solution. Finally, an anti-fluorescence quenching sealing agent (Cat# S2110, Solarbio, Beijing, China) was applied to enable observation under an inverted fluorescence microscope. The additional sections were utilized for TUNEL (Cat# C1088, Beyotime Biotechnology, Shanghai, China) immunofluorescence staining to evaluate apoptosis in the liver following the instructions provided by the reagent manual.

### 2.8 Quantitative PCR analysis

Total RNA was extracted from the fresh liver tissues by using EZ-press RNA Purification Kit (EZBiocience, B0004DP). Subsequently, cDNA synthesis was carried out on the samples using a Colour Reverse Transcription Kit with gDNA Remover (EZBiocience, A0010CGQ) according to the manufacturer’s instructions. Every cDNA sample was diluted with diethylpyrocarbonate (DEPC) water. For PCR amplification, the template was supplemented with 2× Colour SYBR Green qPCR Master Mix (ROX2 plus) (EZBiocience, A0012-R2). The chemical reagents mentioned above were all from ZScience Biotechnology Corporation Limited, San Diego, California, United States. The expression levels of target genes were normalized to the endogenous reference gene glyceraldehyde 3-phosphate dehydrogenase (*Gapdh*) as 2^-△△Ct^. The specific primer sequences are listed in **Table 1**.

**Table 1:** The primer sequences of target genes.

Gene name	Forward primer sequence (5’-3’)	Reverse primer sequence (5’-3’)
Gapdh	ccaaggtcatccatgacaactt	aggggccatccacagtctt
Fasn	tgctgccgtgtccttctact	ttcccatcacacacctggga
Acaca	cgcaccttcttctactggcga	cccagacgtaagccttcactgt
CD36	catgcaagtcctgatgtctcaga	tgaatccagttatgggttccacat
Cpt1a	gacggctatggtgtctcctacat	aagcggtgtgagtctgtctcag
Acox1	ttcgtgcagccagattggtaga	gtggcaatgcgcctcactt
Ppara	aggctatcccaggctttgc	cgtctgactcggtcttttg
Mttp	cgacatcacagtggactcgt	ttgtctcaaattgcctgagtgga
Apob	tgcagttcaaccttgcagtt	tctccctccaagtcccaaag
Tnfa	aaatgggctccctctcatcagttc	tctgcttggtggtttgctacgac
IL-1β	tcgtgctgtctgacccatgt	acaaagctcatggagaataccactt
IL-18	acggagcataaatgaccaagttc	tctgggattcgttggctgtt

### 2.9 Western blotting

Total proteins were extracted from the liver samples as we have previously described (36). The protein concentration was determined by a BCA protein assay kit (Pierce; Thermo Fisher Scientific, Inc.), and a loading amount of 30 µg was used for each sample. Western blotting was performed according to standard procedures (2, 35). The proteins were transferred to polyvinylidene fluoride membranes (Merck Millipore Ltd., Tullagreen, Carrigtwohill, County Cork), which were then incubated with corresponding primary and secondary antibodies by standard techniques. The antibodies against Collagen I (#AF7001), Collagen III (#AF0136), TLR9 (#DF2970), TNF-a (#AF7014), anti-rabbit IgG HRP-linked antibody (# S0001), and anti-mouse IgG HRP-linked antibody (#S0002) were from Affinity Biosciences (Changzhou, Jiangsu, China). The antibodies against TLR4 (#sc-293072), cysteinyl aspartate specific proteinase-3 (Caspase-3, #sc-56053) and Caspase-11 (#sc-374615) were from Santa Cruz Biotechnology (Santa Cruz, CA, USA). The antibodies against phospho-nuclear factor-κB p65 (p-NF-κB p65, #3033S), NF-κB p65 (#8242S), c-Jun NH2-terminal kinase (JNK, #9252S), p-JNK (#9255S) and Caspase-8 (#9746S) were from Cell Signalling Technology (Danvers, MA, USA). The antibodies against 3-nitrotyrosine (3-NT, #ab110282), Caspase-1 (#ab1872), Gasdermin D (GSDMD, #ab219800) and Gasdermin E (GSDME, #ab215191) were from Abcam (Cambridge, UK). The antibody against IL-18 (#D046-3) was from Medical & Biological Laboratories Co., Ltd. (Tokyo, Japan). GAPDH antibody (#MB001) was from Bioworld technology, co. Ltd. (Qixia District, Nanjing, China). The enhanced chemiluminescence (ChemiDoc XRS+ System, Bio-Rad, Hercules, CA, USA) was used to accomplish immunodetection. Western blot bands were quantified in a blinded manner by estimating the integrated density (IntDen) values with Image J software (National Institutes of Health, Bethesda, MD, USA).

### 2.10 Statistical analysis

Statistical analyses were conducted using *one-way analysis of variance* (*ANOVA*) followed by *Bonferroni’s post hoc* test for data showing normal distribution (by *Shapiro-Wilk* test) and homogeneity of variance (by *Brown-Forsythe* test), and performed by *Brown-Forsythe* and *Welch ANOVA* tests followed by *Dunnett T3 post hoc* analysis for data with normal distribution and heteroscedasticity. The *Kruskal-Wallis* test was performed followed by *Dunn’s post hoc* test for data showing non-normal distribution. The “n” in the figures indicates biological replicates. All data were expressed as mean ± SD or median (P25, P75). A value of *p* < 0.05 was considered to indicate statistical significance. All statistical analyses were performed by GraphPad Prism 10.6.0.

## 3 Results

### 3.1 The analysis of active components in Tao-Hong-Si-Wu-Tang water decoction

**Figure 2:**
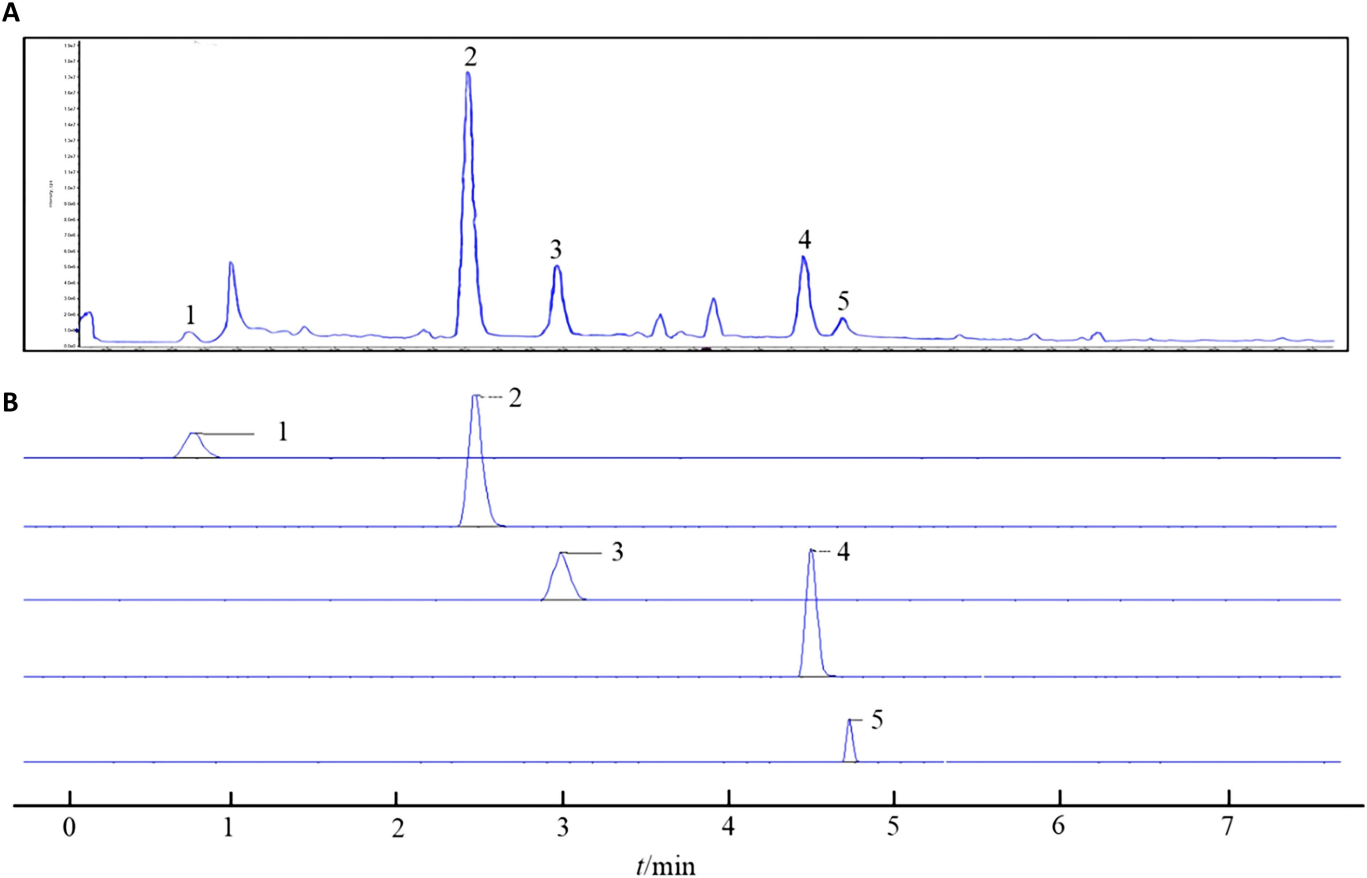
The UPLC chromatograms for content determination of THSWT water decoction **(A)** and reference standards **(B)**. 1, Rehmannioside D; 2, Hydroxysafflor yellow A; 3, Amygdalin; 4, Ferulic Acid; 5. Paeoniflorin.

Based on Chinese Pharmacopoeia (2020 Edition), we performed qualitative analysis of THSWT water decoction according to their corresponding reference substance, including Rehmannioside D, Hydroxysafflor yellow A, Amygdalin, Ferulic Acid and Paeoniflorin. The method validation (using a single-point external standard method) indicated that the calculated concentrations of each reference standard were identical to the target concentrations (**Table 2**). These indicated that the established quantitative method is accurate and reliable, and can be used for the simultaneous quantitative analysis of THSWT water decoction. Based on this, the quantitative analysis of THSWT water decoction showed that each of the five activate components was effectively detected, exhibiting favourable peak shapes and stable retention times. In detail, the retention time of Rehmannioside D is 0.70 min, and its calculated concentration is 70.20 μg/mL; the retention time of Hydroxysafflor yellow A is 2.39 min, and its calculated concentration is 143.00 μg/mL; the retention time of Amygdalin is 2.95 min, and its calculated concentration is 654.00 μg/mL; the retention time of Ferulic Acid is 4.52 min, and its calculated concentration is 76.00 μg/mL; the retention time of Paeoniflorin is 4.70 min, and its calculated concentration is 975.00 μg/mL (**Figure 2** and **Table 3**).

**Table 2:** Quantitative results of reference standards.

Target analyte	Target concentration (μg/mL)	Calculated concentration (μg/mL)	Consistency
Rehmannioside D	51.00	51.00	identical
Hydroxysafflor yellow A	52.50	52.50	identical
Amygdalin	50.00	50.00	identical
Ferulic acid	51.50	51.50	identical
Paeoniflorin	52.50	52.50	identical

**Table 3:** Quantitative results of THSWT water decoction.

Target analyte	Peak area (cps)	Retention time (min)	Test concentration (μg/mL)	Calculated concentration (μg/mL)
Rehmannioside D	1.154e+03	0.70	7.02	70.2
Hydroxysafflor yellow A	4.941e+05	2.39	14.30	143.00
Amygdalin	1.602e+05	2.95	65.40	654.00
Ferulic acid	1.504e+05	4.52	7.60	76.00
Paeoniflorin	1.123e+04	4.70	97.50	975.00

### 3.2 Tao-Hong-Si-Wu-Tang alleviated MCD diet-induced hepatic steatosis in rats

**Figure 3:**
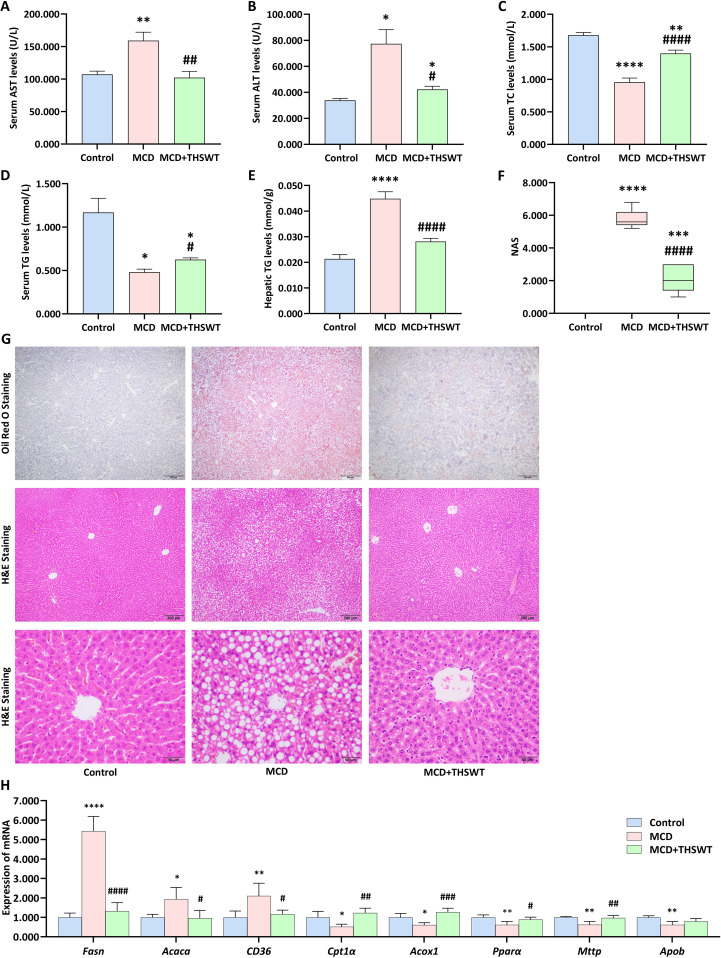
Effects of THSWT on hepatic steatosis induced by feeding a MCD diet in rats. **(A–D)** The serum AST, ALT, TC and TG levels, n = 7 rats in each group. **(E)** Hepatic TG levels, n = 7 rats in each group. **(F)** NAS and **(G)** hepatic oil red O and H&E staining, n = 7 rats in each group. **(H)** The mRNA levels of hepatic lipid metabolism genes, n = 5 rats in each group. The data are expressed as means ± SD. *p < 0.050, **p < 0.010, ***p < 0.001, ****p < 0.0001 vs Control group; ^#^*p* < 0.050, ^##^*p* < 0.010, ^###^*p* < 0.001, ^####^*p* < 0.0001 vs MCD group.

**Figure 4:**
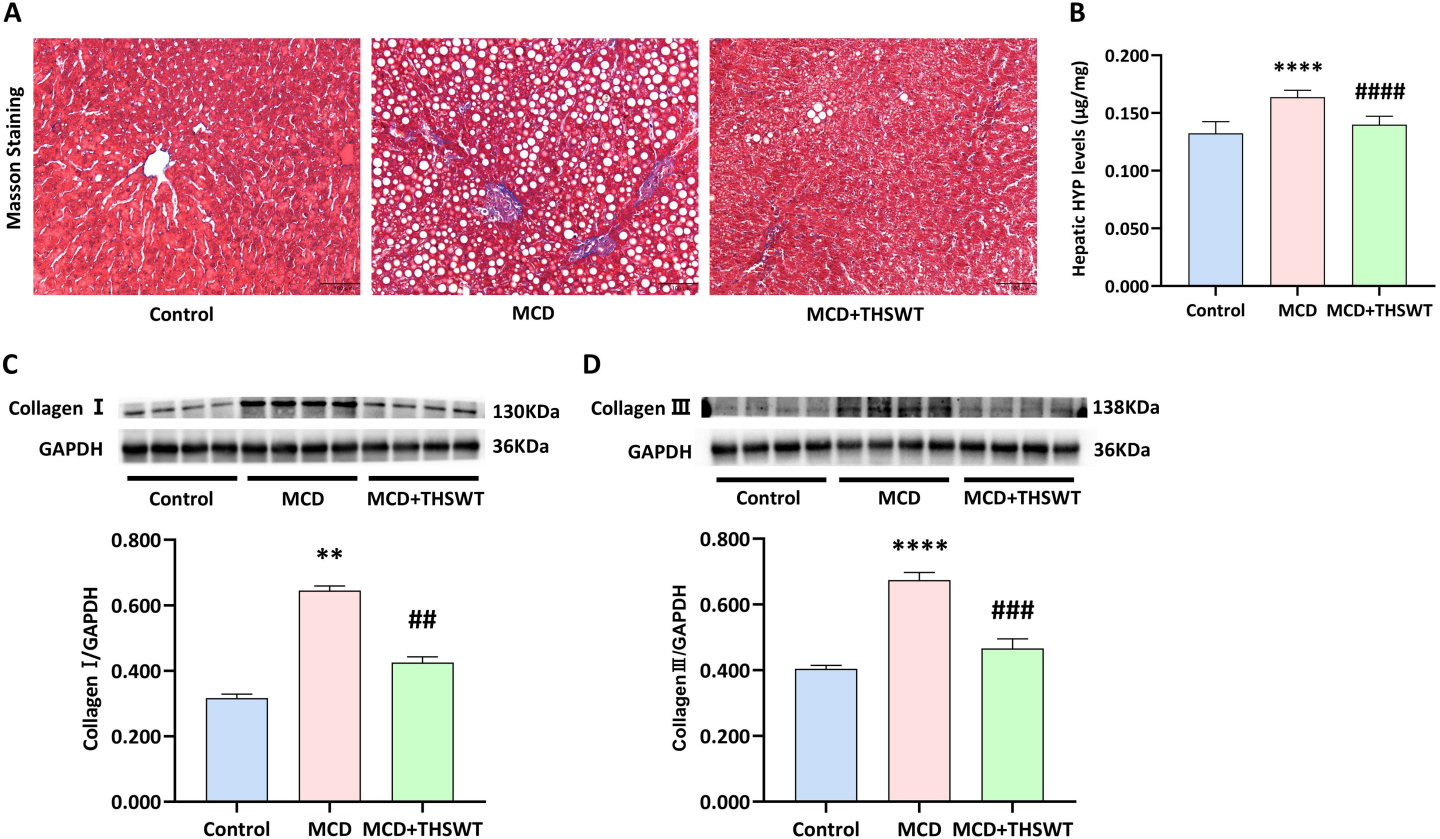
Effects of THSWT on hepatic fibrosis induced by MCD diet in rats. **(A)** Hepatic Masson staining. **(B)** Hepatic hydroxyproline (HYP) levels, n = 7 rats in each group. **(C)** The western blotting images of hepatic collagen I, and quantification of collagen I/GAPDH ratio; **(D)** The western blotting images of hepatic collagen III, and quantification of collagen III/GAPDH ratio, n = 4 rats in each group. Results are means ± SD. **p < 0.010, ****p < 0.0001 vs Control group; ^##^*p* < 0.010, ^###^*p* < 0.001, ^####^*p* < 0.0001 vs MCD group.

The MCD diet feeding contributed to the development of liver injury, as indicated by markedly elevated serum levels of AST and ALT by feeding a MCD diet in rats, while THSWT decreased the up-regulation of serum ALT and AST levels (**Figures 3A, B**). Similar to previous report (31), serum levels of TC and TG were decreased in MCD diet-feeding rats, while these were all increased by THSWT gavage in MCD diet-feeding rats (**Figures 3C, D**). The oil red O staining, H&E staining and NAS, and hepatic TG levels analysis, were further confirmed that THSWT intervention may significantly reduce hepatic steatosis induced by feeding a MCD diet in rats (**Figures 3E–G**). The liver is an essential lipid metabolism organ in the body (37), the above data indicated that lipid metabolism in the liver was damaged, and THSWT may improve these phenotypes. To further confirm this hypothesis, the expression of lipid metabolism genes in the liver were examined by qPCR. Two weeks of MCD-diet feeding in Wistar rats increased mRNA levels of fatty acid synthase (*Fasn*) and acetyl-coenzyme A carboxylase alpha (*Acaca*), which are critical enzymes in *de novo* lipogenesis (DNL) of fatty acid, these were all decreased by THSWT gavage (**Figure 3H**). The mRNA levels of cluster of differentiation 36 (*CD36*, a key gene involved in fatty acid intake) was up-regulated, which was reversed by THSWT treatment (**Figure 3H**). The genes involved in fatty acid oxidation (e.g., carnitine palmitoyl transferase 1 alpha [*Cpt1a*], acyl coenzyme A oxidase 1 [*Acox1*], peroxisome proliferators-activated receptor alpha [*Ppara*]), and fatty acid output (e.g., microsomal triglyceride transfer protein [*Mttp*] and apolipoprotein B [*Apob*]) were all decreased in the liver of MCD diet-fed rats (**Figure 3H**), in contrast, THSWT reversed the down-regulation of these genes, except for no statistically significant effect on *apob* (**Figure 3H**). It has been reported that feeding a MCD diet in a short time can induce slight liver fibrosis (38). We therefore used Masson staining to detect the fibrosis, our data showed that the liver became slight fibrosis in the rats feeding a MCD diet for two weeks (**Figure 4A**), and associated with increasing the hepatic levels of HYP, collagen I and collagen III (**Figures 4B–D**), all these were improved by THSWT. Therefore, THSWT can alleviate liver steatosis and fibrosis induced by MCD diet in rats.

### 3.3 Tao-Hong-Si-Wu-Tang reduced MCD diet-induced hepatic oxidative stress in rats

**Figure 5:**
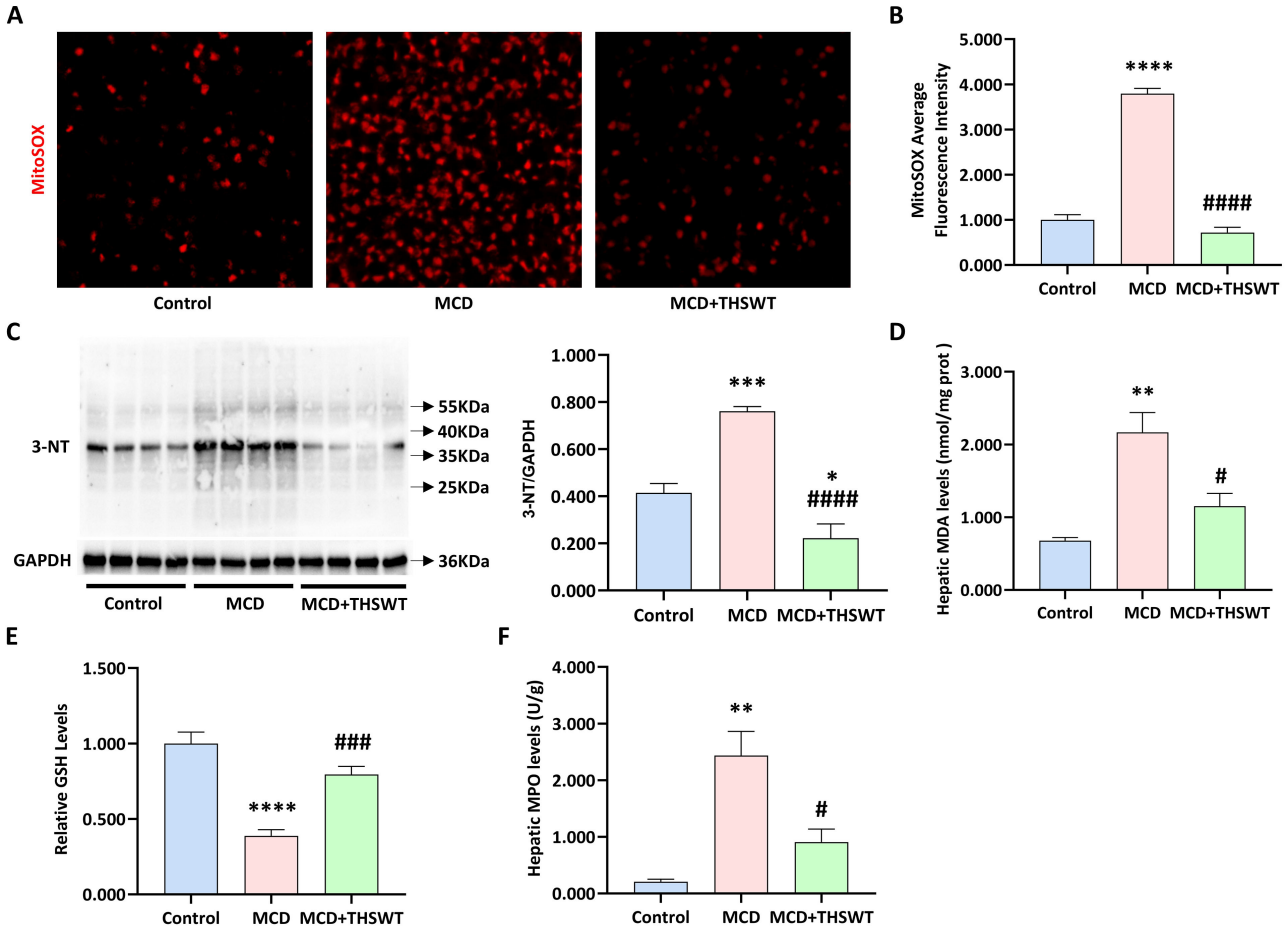
Effects of THSWT on hepatic oxidative stress induced by MCD diet in rats. **(A)** The MitoSOX immunofluorescence staining. **(B)** MitoSOX average fluorescence intensity (the average of the Control was set as “1”), n = 4 rats in each group. **(C)** The western blotting images of hepatic 3-NT and GAPDH, and quantification of 3-NT/GAPDH ratio, n = 4 rats in each group. **(D)** The hepatic MDA levels, n = 7 rats in each group. **(E)** The hepatic GSH levels, n = 7 rats in each group. **(F)** The hepatic MPO levels (the average of Control was set as "1"), n = 7 rats in each group. Results are means ± SD. *p < 0.050, **p < 0.010, ***p < 0.001, ****p < 0.0001 vs Control group; ^#^p < 0.050, ^###^*p* < 0.001, ^####^*p* < 0.0001 vs MCD group.

The rats or mice feeding a MCD diet is strongly associated with hepatic oxidative stress, which contributed to the development of NAFLD (39–42). In order to investigate anti-oxidative effects of THSWT, the production of oxidative stress markers (ROS, 3-NT and MDA) and consumption of anti-oxidants (GSH) in the liver were evaluated. Hepatic mitochondrial ROS (mitoSOX), 3-NT and MDA levels were increased in MCD-feeding rats (**Figures 5A–D**), on the contrary, GSH levels in the liver were decreased (**Figure 5E**). Treatment with THSWT decreased mitochondrial ROS, 3-NT, and MDA levels in the liver, while increase hepatic GSH levels (**Figures 5A–E**). Oxidative stress can also induce neutrophils infiltrations to promote myeloperoxidase (MPO) accumulations, which further induces oxidative stress and inflammation (43, 44). Similarly, THSWT also decreased hepatic MPO levels induced by feeding a MCD diet in rats (**Figure 5F**). Therefore, THSWT dramatically reduced MCD diet-induced hepatic oxidative stress in rats.

### 3.4 Tao-Hong-Si-Wu-Tang inhibited TLR4/9-mediated innate immune signalling in MCD diet-fed rats

**Figure 6:**
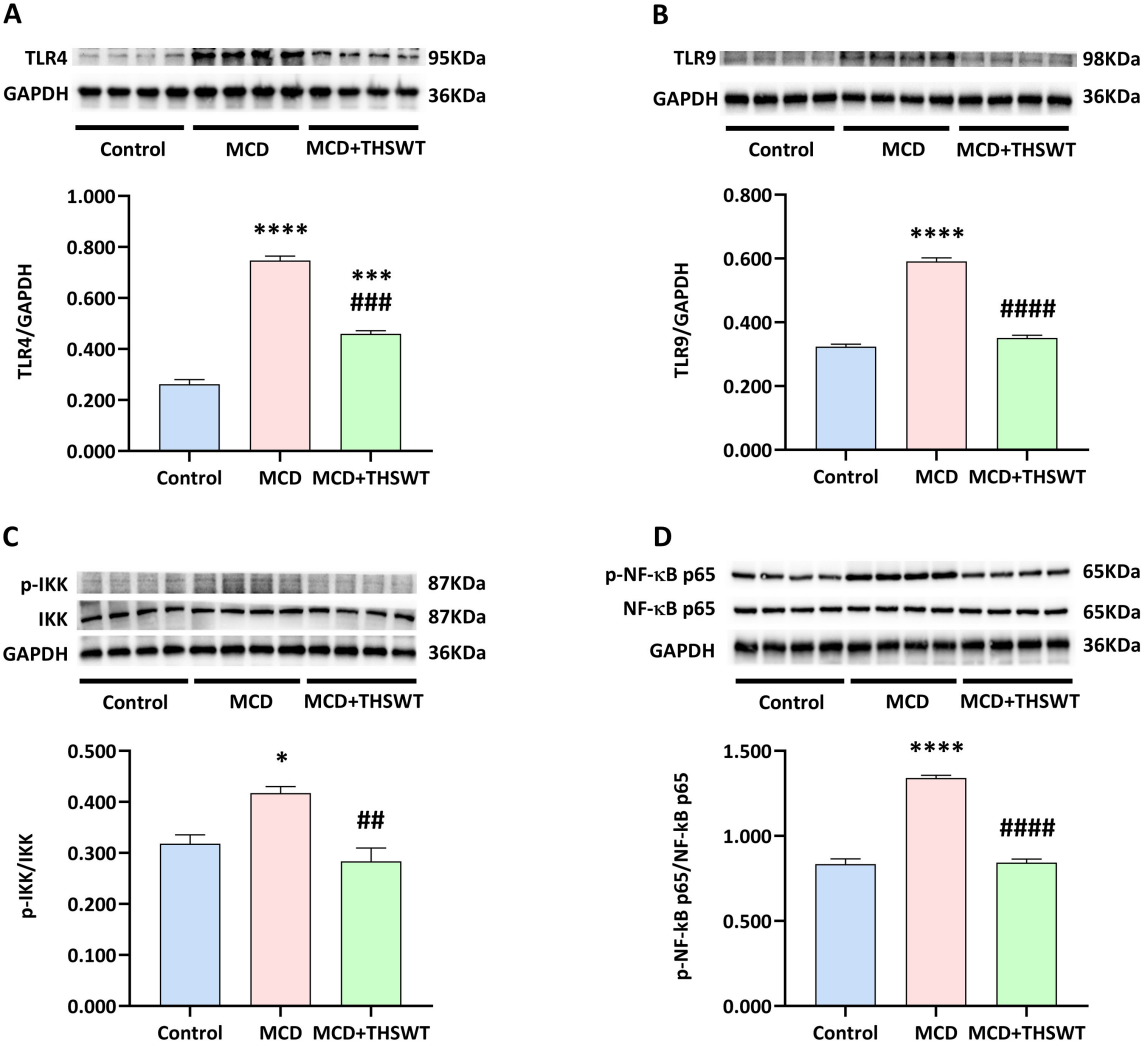
Effects of THSWT on hepatic TLR4/9-mediated innate immune signalling and expression of inflammatory cytokines induced by MCD diet in rats. **(A–D)** The western blotting images of hepatic TLR4, TLR9, p-IKK, IKK, p-NF-κB p65, NF-κB p65 and their corresponding GAPDH, and quantifications of TLR4/GAPDH, TLR9/GAPDH, p-IKK/IKK, and p-NF-κB p65/NF-κB p65 ratios, n = 4 rats in each. Results are means ± SD. *p < 0.050, ***p < 0.0010, ****p < 0.0001 vs control group; ^##^p < 0.01, ^###^*p* < 0.001, ^####^*p* < 0.0001 vs MCD group.

**Figure 7:**
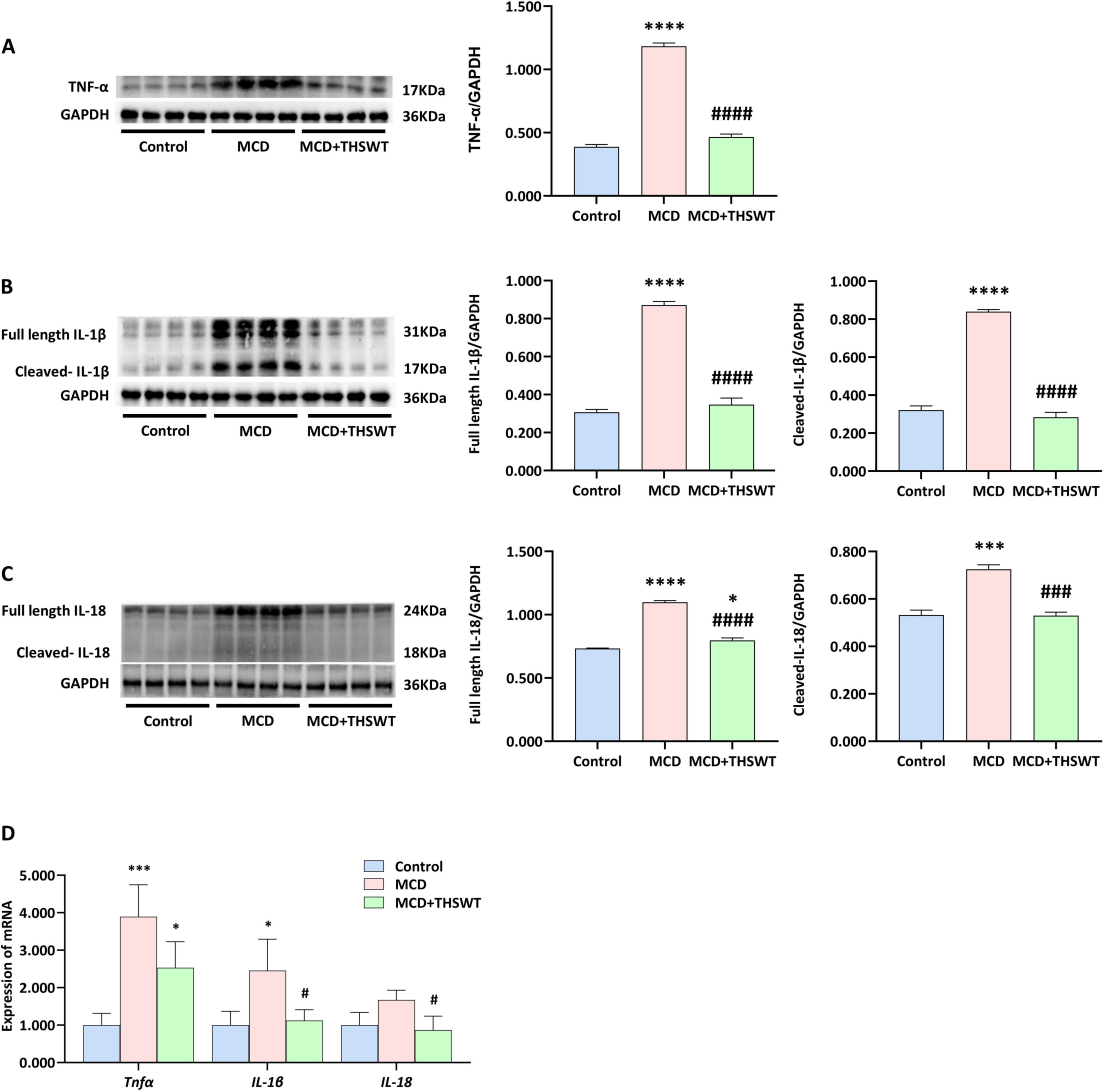
Effects of THSWT on hepatic inflammatory cytokines expression induced by MCD diet in rats. **(A–C)** The western blotting images of hepatic TNF-α, IL-1β, IL-18 and their corresponding GAPDH, and quantifications of TNF-α/GAPDH, full length IL-1β/GAPDH, cleaved-IL-1β/GAPDH, full length IL-18/GAPDH, and cleaved-IL-18/GAPDH ratios, n = 4 rats in each. **(D)** The mRNA levels of inflammatory cytokines in the liver, n = 4 rats in each group. Results are means ± SD. *p < 0.050, ***p < 0.001, ****p < 0.0001 vs Control group; ^#^p < 0.05, ^###^*p* < 0.001, ^####^*p* < 0.0001 vs MCD group.

Over-activation of TLR4/9-mediated NF-κB innate immune signalling plays an important role in the pathogenesis of NAFLD (45, 46). Deficiency of TLR4, or its coreceptor, myeloid differentiation factor-2 (MD-2) attenuates NASH and fibrosis in mice received MCD diet (45). TLR9 promotes steatohepatitis by induction of IL-1β in mice fed a choline-deficient amino acid-defined (CDAA) diet (46). Therefore, we examined the expression of TLR4 and TLR9, and the activation of downstream transcription factor NF-κB and the expression of pro-inflammatory cytokines. Our results showed the expression of hepatic TLR4 and TLR9, the phosphorylation of hepatic NF-κB p65, and the protein and mRNA levels of hepatic TNF-α, IL-1β, and IL-18 were all up-regulated by feeding a MCD diet in rats, all these were suppressed by treatment with THSWT (**Figures 6**, **7**). Therefore, THSWT can attenuate TLR4/9-NF-κB innate immune signalling mediated pro-inflammatory cytokines expression.

### 3.5 Tao-Hong-Si-Wu-Tang inhibited hepatic apoptosis signalling in MCD diet-fed rats

**Figure 8:**
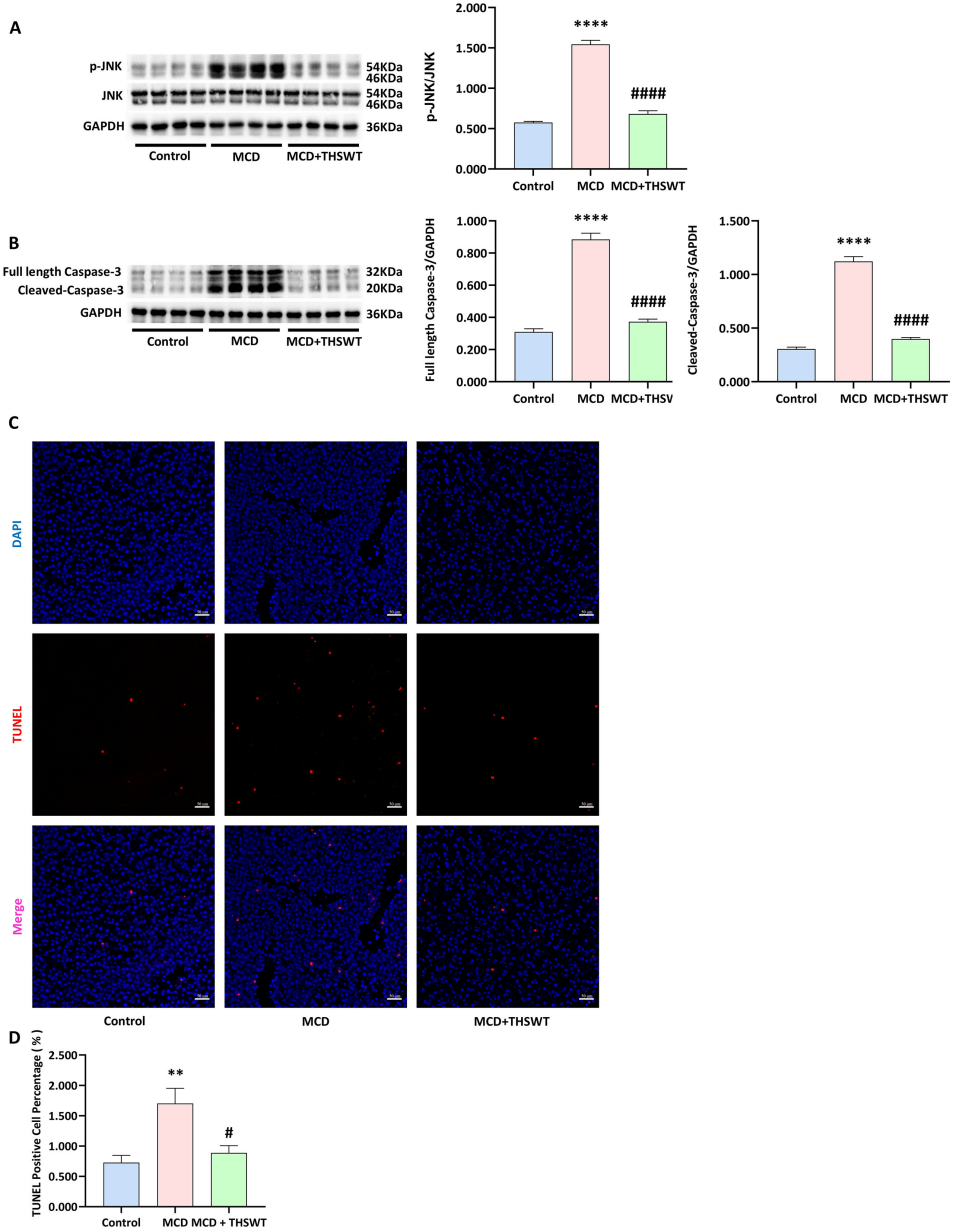
Effects of THSWT on hepatic apoptosis induced by feeding a MCD diet in rats. **(A)** The western blotting images of hepatic p-JNK, JNK and GAPDH, and quantification of p-JNK/JNK ratio, n = 4 rats in each group. **(B)** The western blotting images of hepatic full-length Caspase-3, cleaved-Caspase-3 and GAPDH, and quantifications of full-length Caspase-3/GAPDH and cleaved-Caspase-3/GAPDH ratios, n = 4 rats in each group. **(C)** The TUNEL immunofluorescence staining and **(D)** TUNEL positive cell percentage, n = 4 rats in each group. Results are means ± SD. **p < 0.010, ****p < 0.0001 vs Control group; ^#^p < 0.05, ^####^*p* < 0.0001 vs MCD group.

Besides increasing pro-inflammatory cytokines expression *via* the classical transcription factor NF-κB, TLR4/9 signalling can also activate pro-apoptotic signalling cascade involving jun N-terminal kinase (JNK) (47, 48). Therefore, we investigated whether THSWT can inhibit apoptosis induced by feeding a MCD diet in rats. The WB data showed THSWT decreased JNK phosphorylation (**Figure 8A**) and reduced both the full-length and cleaved-forms of Caspase-3 in the liver (**Figure 8B**). TUNEL immunofluorescence staining indicated that the percentage of apoptosis-positive cells were increased in rats by feeding a MCD diet, and this was reduced by treatment with THSWT (**Figures 8C, D**). Therefore, these indicated that THSWT inhibited hepatic apoptosis in MCD diet-fed rats.

### 3.6 Tao-Hong-Si-Wu-Tang suppressed hepatic pyroptosis signalling in MCD diet-fed rats

**Figure 9:**
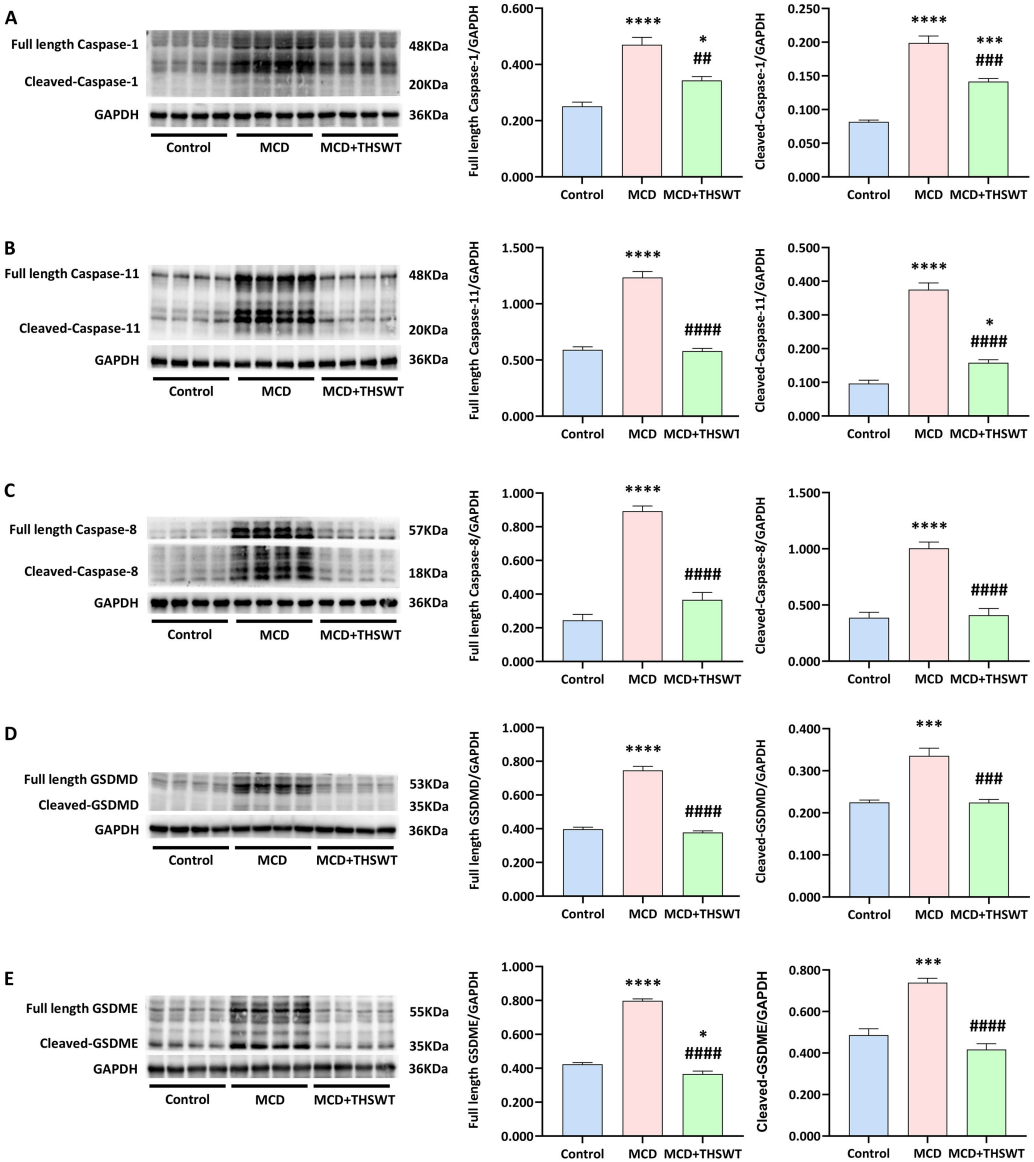
Effects of THSWT on hepatic pyroptosis induced by feeding a MCD diet in rats. **(A–E)** The western blotting images of hepatic full-length and cleaved Caspase-1, Caspase-11, Caspase-8, GSDMD, and GSDME, and their corresponding GAPDH, and quantifications of these proteins to their corresponding GAPDH, n = 4 rats in each. Results are means ± SD. *p < 0.050, ***p < 0.001, ****p < 0.0001 vs Control group; ^##^*p* < 0.01, ^###^*p* < 0.001, ^####^*p* < 0.0001 vs MCD group.

The pro-IL-1β and pro-IL-18 were cleaved and matured by the activated Caspase-1 (cleaved-Caspase-1) (49), and the cleaved Caspase-1, Caspase-11, and Caspase-8 can also trigger common substrate protein GSDMD to induce pyroptosis (50–52). Therefore, to further investigate whether THSWT can inhibit pyroptosis induced by MCD diet in rats, we examined hepatic pyroptosis signalling. The protein levels of full-length Caspase-1, cleaved-Caspase-1, full-length Caspase-11, cleaved-Caspase-11, full-length Caspase-8, and cleaved-Caspase-8 were increased in the liver of MCD diet-fed rats, while these were all inhibited by THSWT (**Figures 9A–C**). Besides GSDMD, GSDME can also be cleaved by Caspase-3, and further induce pyroptosis (53). We finally examined the activation of GSDMD and GSDME. Our data showed that hepatic full-length-GSDMD, cleaved-GSDMD, full-length-GSDME and cleaved-GSDME were all inhibited by THSWT gavage in MCD-fed rats (**Figure 9D, E**). Therefore, THSWT suppressed hepatic pyroptosis signalling in MCD diet-fed rats.

## 4 Discussion

THSWT is an ancient TCM prescription recorded in “*Yi Zong Jin Jian*” and now is included in the list of ancient famous medicines by the State Administration of TCM. It is a classic blood tonifying formula based on Si-Wu-Tang (SWT; includes Shudi (*Rehmannia glutinosa* (Gaertn.) Libosch.), Chuanxiong (*Ligusticum chuanxiong* Hort.), Baishao (*Paeonia lactiflora* Pall.), Danggui [*Angelica sinensis* (Oliv.) Diels.)], with the addition of Taoren (*Prunus persica* (Linn) Batsch.), and Honghua (*Carthamus tinctorius* L.). Previously, we showed that SWT can alleviate MCD diet-induced NAFLD in mice (2). Oral administration of SWT for 6 months improved the antioxidant level and positively regulated the lipid profile, liver function, and skin integrity and texture in healthy adults (54). THSWT also exhibited protective effects on the liver, as that it alleviated thioacetamide- or carbon tetrachloride (CCl_4_)-induced liver fibrosis in mice (27–30, 55), and reduced hepatic lipid accumulation in female mice with heart failure with preserved ejection fraction (HFpEF) (26). Here, we showed that THSWT significantly improved hepatic steatosis, reduced serum ALT and AST levels, while increased serum TC and TG levels in rats feeding a MCD diet. The upregulation of serum TC and TG levels by THSWT is associated with increasing lipid export gene *Mttp* expression, however, its long-term effects (beneficial or harmful) on blood lipid regulation still need further investigation. Rehmannioside D (56), Hydroxysafflor yellow A (57), Amygdalin (58), Ferulic Acid (59), and Paeoniflorin (60) all exhibited liver-protective effects. Whether these ingredients are the material basis for THSWT on NAFLD still needs to be verified by analysis of the active components in serum combined with cellular experiments. Furthermore, considered that MCD diet may cause weight loss and do not induce insulin resistance (2), therefore, high-fat diet (HFD) should be used to further compare the therapeutic effects of these two formulas on animals of the same strain, and explore the advantages and disadvantages of liver-protective effects of these two classic formulas.

The liver is a crucial organ of lipid metabolism, it participates and regulates four key events as *de novo* lipogenesis, fatty acid uptake, fatty acid oxidation and lipid export (37). Here, we showed that MCD feeding increased the mRNA levels of DNL genes *Acaca* and *Fasn*, and fatty acid uptake gene *CD36* in the liver; decreased the mRNA levels of fatty acid oxidation related genes *Cpt1*, *Acox1*, and *Ppara*, and lipid export genes *Mttp* and *Apob* in the liver of rats. THSWT treatment can reverse these genes levels, excluding *Apob*. These indicated that THSWT can regulate hepatic lipidmetabolism in this animal model. Moreover, SWT and Taoren-Honghua herb pair alleviated hepatic inflammation and fibrosis induced by CCl4 (55, 61). Our data showed that THSWT was also able to improve MCD diet-induced mild hepatic fibrosis in rats, and down-regulate hepatic Collagen I and III protein levels. Therefore, the above data indicated that THSWT has a therapeutic effect on NAFLD induced by feeding a MCD diet in rats.

In addition to reduced fatty acid oxidation in the liver, hepatic mitochondrial ROS (mtROS) was increased with increasing NAFLD severity and hepatic fibrosis in humans with obesity (62). The increased ROS can also contribute to the up-regulation of 3-NT (the product of protein tyrosine residues nitration) and MDA (a form of lipid peroxidation product), and the consumption of GSH (antioxidant) (63, 64), which further aggravates oxidative damage (65). Here, the MCD feeding induced a mass accumulation of hepatic mtROS, 3-NT and MDA levels, and a large consumption of hepatic GSH levels in the liver. THSWT has an anti-oxidation effect (66), and due to containing multiple bioactive components with anti-oxidation, such as ferulic acid (67), hydroxysafflor yellow A (68), gallic acid (69), senkyunolide I (70), and paeoniflorin (71). Our results suggested that THSWT decreased hepatic mtROS, 3-NT and MDA levels while increased hepatic GSH levels. Neutrophil activation also releases ROS (72). Neutrophil infiltration is one of the hallmarks of NASH around lipotoxic hepatocytes (73). Neutrophil releases MPO to exacerbate liver lipid accumulations, inflammation, and fibrosis in NASH mice model induced by HFD (74). In contrast, congenital absence of MPO can improve MCD diet-induced liver injury and fibrosis in mice (75). We demonstrated that THSWT can reduce hepatic MPO levels in NAFLD rats. In general, THSWT inhibited hepatic oxidative stress induced by feeding a MCD diet in rats.

The mROS can be triggered by coupling TLRs (e.g., TLR1, TLR2 and TLR4) signalling to mitochondrial Complex I through TRAF6 (tumour necrosis factor receptor-associated factor 6) and ECSIT (evolutionarily conserved signalling intermediate in Toll pathways) (76). Hepatic macrophages generate ROS in response to palmitate through NADPH oxidase 2 *via* endocytosis of monomeric TLR4-MD2 complex (77). Similarly, loss of TLR9 decreased ROS levels concomitant with a high bacterial load in the macrophages and in the livers of TLR9^-/-^ mice (78). Moreover, TLR9 deficiency improved hepatic steatosis, inflammation, and fibrosis in mice fed with a choline-deficient amino acid-defined (CDAA) diet (46). Similarly, TLR4 deficiency improved NAFLD in mice (79). Lipopolysaccharides (LPS; an agonist of TLR4) serum levels and LPS hepatocyte localization were increased in NASH patients and mice compared with their corresponding controls, which were correlated with NF-κB activation (80). Besides generating ROS, TLR4/9-mediated innate immune signalling classically activates NF-κB signalling, which contribute to the expression of proinflammatory cytokines, and thus amplifying inflammation (81, 82). Our results showed that hepatic TLR4 and TLR9 levels, the phosphorylation of downstream signalling IKK and NF-κB, and the inflammatory cytokines (TNF-α, IL-1β, and IL-18) were increased after feeding a MCD diet in rats, all these were reversed by THSWT treatment. TLRs innate immune signalling can also activate MAPKs, which are essential contributors of NAFLD (81, 83). The suppressor of innate immune signalling, such as suppressor of IKKϵ (SIKE), deubiquitinase TNFα-induced protein 3 (TNFAIP3), and cylindromatosis (CYLD), has been emerged as key targets for treating NAFLD (84–86). However, the actions of THSWT on MAPKs, and the innate immune inhibitors are unclear.

Besides NF-κB, TLR4 can also initiate a TRAF6-JNK-mediated cell death pathway that is upstream of Caspase activation (47). Apoptosis is triggered by the apoptotic Caspases, Caspases-8, Caspases-9 and Caspases-10 initiate apoptosis through the activation of the executioner Caspases-3, Caspases-6 and Caspases-7 (87). For example, TNF-α induced hepatocyte apoptosis *via* activating Caspase-8 and Caspase-3 (88). THSWT has the anti-apoptosis effect on osteocytes (25). Here, western blotting of JNK and Caspase-3 and Caspase-8, and TUNEL staining were used to confirm the effect of THSWT on apoptosis. Our data showed that THSWT can inhibit hepatic apoptosis induced by feeding a MCD diet in rats.

Besides apoptosis, pyroptosis also plays an essential role in the pathogenesis of NAFLD (19, 89). The Caspase-1 to GSDMD signalling is the classical pyroptosis pathway (50), which is classically activated by inflammasome, such as NLRP3 inflammasome (which is a complex of NLRP3, the adaptor protein ASC, and Caspase-1) (90). The activated Caspase-1 contribute to maturating pro-IL-1β and pro-IL-18 to amplify inflammation, and inducing pyroptosis by cleavage of GSDMD (91). Our data showed that the full-length and cleaved forms of Caspase-1 and GSDMD in the liver were increased by MCD feeding in rats, which were reversed by THSWT. Pyroptosis can also be triggered by the non-classical Caspase-4/5/11-GSDMD signalling and Caspase-11 can be directly recognised by LPS (51, 92). Caspase-4/5 exists in human and their homologous Caspase-11 exists in rodents (92). Caspase-8 can also induce cleavage of GSDMD to elicit pyroptosis (93). The full-length and cleaved fragments of hepatic Caspase-11 and Caspase-8 were increased in rats fed with a MCD diet, and THSWT treatment can decrease these up-regulation. GSDME is another pyroptosis trigger specifically cleaved by Caspase-3 in its linker (53). We found that both full-length and cleaved fragments of hepatic GSDME were increased in MCD diet feeding rats, THSWT can reduce the full-length and cleaved fragments of hepatic GSDME induced by MCD diet feeding in rats. Overall, THSWT inhibited hepatic pyroptosis induced by MCD diet feeding in rats.

## 5 Conclusions

**Figure 10:**
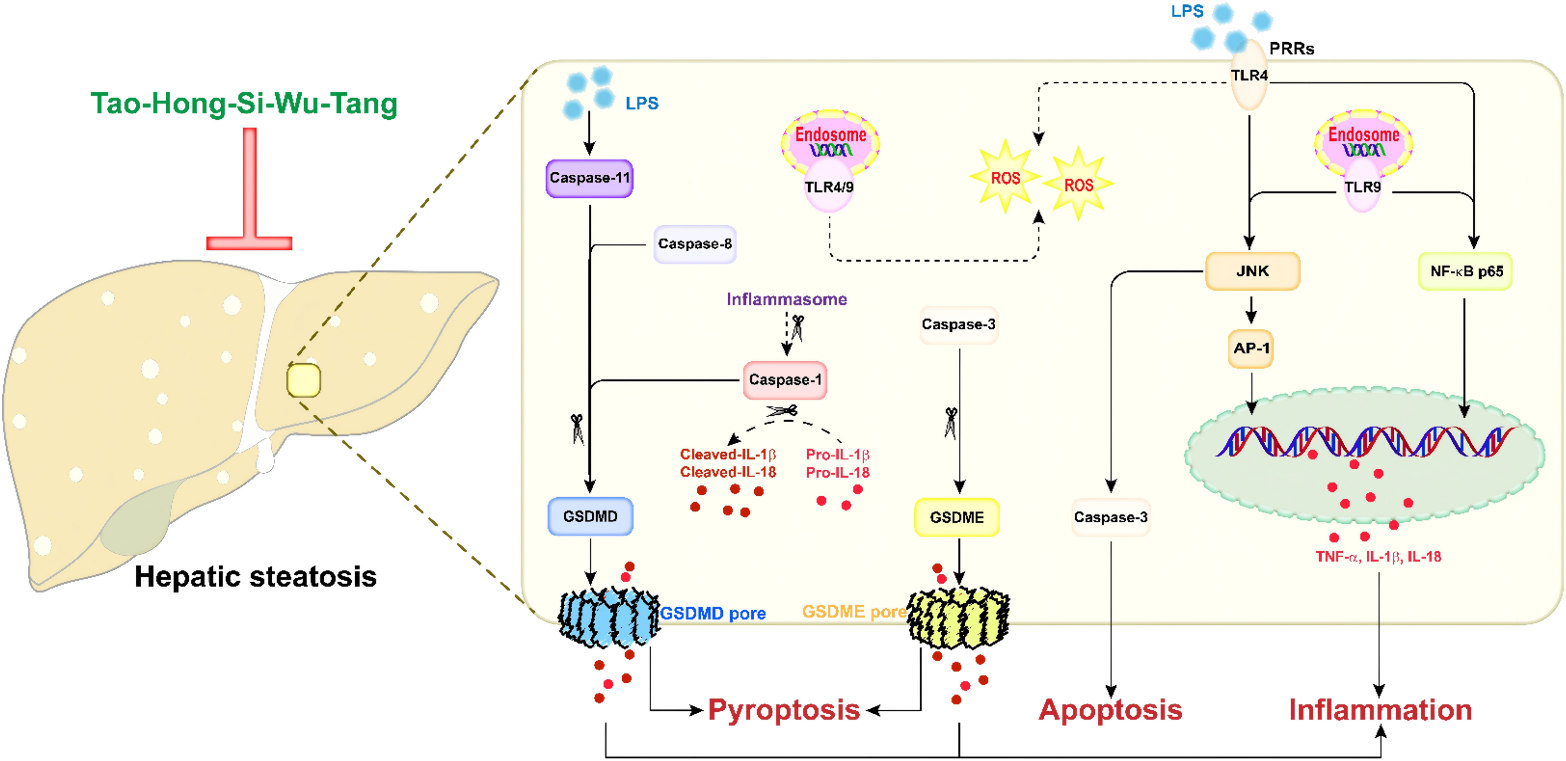
Tao-Hong-Si-Wu-Tang protects against MCD diet-induced hepatic steatosis in rats and is associated with the inhibition of TLR4/9-NF-κB innate immune signalling, oxidative stress, Caspase-3-mediated apoptosis signalling, Caspase-1/11/8 to GSDMD, and Caspase-3 to GSDME pyroptosis signalling in the liver.

The ancient TCM formula THSWT alleviated MCD diet-induced hepatic steatosis in rats and was associated with inhibition of TLR4- and TLR9-mediated innate immune signalling, apoptosis, and GSDMD- and GSDME-mediated pyroptosis in the liver (**Figure 10**).

## Data Availability

The raw data supporting the conclusions of this article will be made available by the authors, without undue reservation.

## Glossary

NAFLD: Non-alcoholic fatty liver disease
MASLD: Metabolic dysfunction-associated steatotic liver disease
NAFL: Non-alcoholic fatty liver
NASH: Non-alcoholic steatohepatitis
HCC: Hepatocellular carcinoma
CVDs: Cardiovascular diseases
TCM: Traditional Chinese medicine
THSWT: Tao-Hong-Si-Wu-Tang
HPLC: High-performance liquid chromatography
MCD: Methionine- and choline-deficiency
H&E: Hematoxylin and eosin
ALT: Alanine aminotransferase
AST: Aspartate aminotransferase
TC: Total cholesterol
TG: Triglycerid
MDA: Malondialdehyde
GSH: Glutathione
MPO: Myeloperoxidase
ROS: Reactive oxygen species
q-PCR: Quantitative Real-time polymerase chain reaction
DEPC: Diethylpyrocarbonate
Gapdh: Glyceraldehyde 3-phosphate dehydrogenase
TLR9: Toll-like receptor 9
TNF-α: Tumour necrosis factor alpha
TLR4: Toll-like receptor 4
Caspase-3: Cysteinyl aspartate specific proteinase-3
Caspase-11: Cysteinyl aspartate specific proteinase-11
p-NF-κB p65: Phospho-nuclear factor-kappa B p65
NF-κB p65: Nuclear factor-kappa B p65
JNK: c-Jun N-terminal kinase
P-JNK: Phosphorylated c-Jun N-terminal kinase
Caspase-8: Cysteinyl aspartate specific proteinase-8
3-NT: 3-nitrotyrosine
IL-1β: Interleukin-1 beta
Caspase-1: Cysteinyl aspartate specific proteinase-1
GSDMD: Gasdermin D
GSDME: Gasdermin E
IL-18: Interleukin-18
Fasn: Fatty acid synthase
Acaca: Acetyl-coenzyme A carboxylase alpha
DNL: *De novo* lipogenesis
CD36: Cluster of differentiation 36
Cpt1a: Carnitine palmitoyl transferase 1 alpha
Acox1: Acyl coenzyme A oxidase 1
Ppara: Peroxisome proliferators-activated receptor alpha
Mttp: Microsomal triglyceride transfer protein
Apob: Apolipoprotein B
MD-2: Myeloid differentiation factor-2
CDAA: Choline-deficient amino acid-defined
SWT: Si-Wu-Tang
CCl4: Carbon Tetrachloride
HFD: High-fat diet
mtROS: mitochondrial ROS
TLR1: Toll-like receptors 1
TLR2: Toll-like receptors 2
TRAF6: Tumour necrosis factor receptor-associated factor 6
ECSIT: Evolutionarily conserved signalling intermediate in Toll pathways
LPS: Lipopolysaccharides
IL-6: Interleukin-6
NLRP3: NLR Family Pyrin Domain Containing Protein 3
